# Chronic Pain in the Emergency Department: A Pilot Interdisciplinary Program Demonstrates Improvements in Disability, Psychosocial Function, and Healthcare Utilization

**DOI:** 10.1155/2018/1875967

**Published:** 2018-01-17

**Authors:** Joshua A. Rash, Patricia A. Poulin, Yaadwinder Shergill, Heather Romanow, Jeffrey Freeman, Monica Taljaard, Guy Hebert, Ian G. Stiell, Catherine E. Smyth

**Affiliations:** ^1^Department of Psychology, Memorial University of Newfoundland, St. John's, NL, Canada; ^2^The Ottawa Hospital Research Institute, Ottawa, ON, Canada; ^3^School of Psychology & Department of Anesthesiology, University of Ottawa, Ottawa, ON, Canada; ^4^The Ottawa Hospital Department of Psychology, Ottawa, ON, Canada; ^5^Department of Emergency Medicine, University of British Columbia, Vancouver, BC, Canada; ^6^School of Epidemiology, Public Health and Preventive Medicine, University of Ottawa, Ottawa, ON, Canada; ^7^Department of Emergency Medicine, Faculty of Medicine, University of Ottawa, Ottawa, ON, Canada

## Abstract

**Objective:**

To evaluate the feasibility of an individualized interdisciplinary chronic pain care plan as an intervention to reduce emergency department (ED) visits and improve clinical outcomes among patients who frequented the ED with concerns related to chronic pain.

**Methods:**

A prospective cohort design was used in an urban tertiary care hospital. As a pilot program, fourteen patients with chronic pain who frequented the ED (i.e., >12 ED visits within the last year, of which ≥50% were for chronic pain) received a rapid interdisciplinary assessment and individualized care plan that was uploaded to an electronic medical record system (EMR) accessible to the ED and patient's primary care provider. Patients were assessed at baseline and every three months over a 12-month period. Primary outcomes were self-reported pain and function assessed using psychometrically valid scales.

**Results:**

Nine patients completed 12-month follow-up. Missing data and attrition were handled using multiple imputation. Patients who received the intervention reported clinically significant improvements in pain, function, ED visits, symptoms of depression, pain catastrophizing, sleep, health-related quality of life, and risk of future aberrant opioid use.

**Discussion:**

Individualized care plans uploaded to an EMR may be worth implementing in hospital EDs for high frequency visitors with chronic pain.

## 1. Introduction

The Pareto principle [1], as applied to health care, is exhibited in the tremendous use of resources by a small percentage of users. For example, while comprising just 4.5%–8% of patients, high frequency visitors (HFVs) of the emergency department (ED) (definitions vary from ≥3 to ≥12 visits per year) account for 21%–28% of all visits [2]. HFVs of the ED have been characterized as having psychosocial challenges, chronic medical conditions, and low socioeconomic status [3]. While heterogeneity among HFVs of the ED has been reported [2], pain is ubiquitous across medical diagnoses, mental health disorders, and socioeconomic states and may represent a common factor underlying ED visits by HFVs [4]. Indeed, chronic pain was reported as the most common chief complaint among patients with ≥6 annual visits to the ED at a large urban centre [5], and 36% of patients with ≥12 annual ED visits at our center reported chronic pain as the driving factor [6].

Many pain complaints are nonemergent and inappropriate for the ED [7, 8]. This is generally the case for chronic pain, defined as recurrent or persistent pain lasting for more than 3 to 6 months or beyond normal duration of healing [9]. Chronic pain is best managed in the primary care environment [10] using a chronic disease model [11]. Despite this, patients with chronic pain account for 11–16% of visits to the ED [4, 11, 12], with 7% of these patients visiting multiple times per year [13].

Acute exacerbations of chronic pain can be costly [14], and focusing healthcare reform on a small number of HFVs who present with chronic pain may reduce overall costs and yield rapid improvements in treatment outcomes [15]. Inappropriate use of the ED for nonemergent conditions, such as chronic pain, increases the risk for a multitude of operational and care-related outcomes, including overcrowding [16, 17], increased waiting times [18], negative effects on patients, staff and caregiver satisfaction [19], and increased risk of subsequent adverse events [5, 20–22]. Targeting healthcare reform on HFVs who present with chronic pain may also improve the current opioid crisis in North America, where governments, healthcare systems, and communities are looking for methods to ameliorate the 200%–500% rise in opioid consumption and nearly 4-fold increase in opioid-related mortality that occurred from 1999 to 2011 [23].

Reviews of interventions to reduce ED visits among HFVs have reported that interdisciplinary case management and individualized care plans have modest effects with reduced ED visits and associated costs [24–27]. Few studies have specifically focused on chronic pain where there is reason to believe that interdisciplinary approaches could be particularly effective. Several well-conducted studies have concluded that interdisciplinary pain management programs result in global benefits when compared to numerous other common pain management interventions, including medication, surgery, and cognitive behavior therapy [28–31]. Moreover, evidence-based clinical practice guidelines for the treatment of chronic pain outside of the ED recommend the use of interdisciplinary pain management programs with “strong” supporting evidence (e.g., [32, 33]). It stands to reason that interdisciplinary individualized chronic pain care plans would result in global improvements for HFVs attending the ED with chronic pain.

This investigation evaluated the feasibility of an individualized interdisciplinary chronic pain care plan developed in conjunction with the hospital emergency team, primary care provider (PCP), and patient to improve pain, healthcare utilization, and clinical outcomes endorsed by the Initiative on Methods, Measurement, and Pain Assessment in Clinical Trials (IMMPACT) [34]. The aim was to determine if an interdisciplinary program linked with the emergency department and primary care was feasible and if it would result in improved clinical outcomes and reduced emergency room visits.

## 2. Methods

### 2.1. Design

This was a 12-month prospective cohort design. Patients were identified, underwent baseline evaluation, received intervention, and were assessed at 3-month intervals over a subsequent 12-month follow-up period. Data obtained at 12-month follow-up were compared to baseline data among the cohort.

### 2.2. Setting

The study was conducted at a large, urban, tertiary care hospital with 172,445 patient visits per year.

### 2.3. Participants

#### 2.3.1. Inclusion Criteria

Patients were referred by ED physicians and screened by a member of the research team with significant experience in the management of chronic pain (CS, PP or YS) to verify whether they met the following inclusion criteria: (1) over 18 years of age; (2) experienced pain that persisted longer than 3 months; (3) were high frequency visitors of the ED, defined as visiting the ED 12 or more times in the previous 12 months; (4) ≥50% of ED visits were for chronic pain; and (5) spoke English or French. Inability to provide consent or active psychosis were exclusion criteria for this study.

The study protocol was reviewed by the Chair of the institutional REB and approved as a quality improvement initiative. All participants underwent an individual consent process and provided written consent to participate. Informed consent was revisited during 3-, 6-, 9-, and 12-month visits.

### 2.4. Procedures

Consenting patients received rapid (<2 weeks) access to a comprehensive assessment by the Rapid Interprofessional Pain Assessment Program (RIPAP) team. Composition of the RIPAP team depended on patients' needs identified through a chart review completed by a pain specialist and consultation with the referring ED physician. At a minimum, the RIPAP team comprised a physician with focused expertise in pain management, nurse, social worker, and health psychologist. Patients who screened positive for risk of opioid abuse as a result of a high score on the opioid risk tool administered during the clinical interview and/or signs of opioid aberrancies (requests for early prescription) were also referred to addiction medicine. Results from the comprehensive assessment and subsequent treatment recommendations were discussed with the patient to engage them in decisions about their healthcare and ensure that their values, preferences, and goals were reflected in a comprehensive treatment plan. The treatment plan was sent to PCPs and ED physicians with whom outpatient pain case conferences were convened, and input to the plan was concurrently sought. The finalized treatment plan served as an individualized chronic pain care plan that detailed the approach that would be taken to assist the patients in managing their pain on an outpatient basis, as well as recommendations for the management of pain in the event of further ED visits. The care plan was uploaded to the hospital's electronic medical record system; refer to supplementary file for a sample care plan. Patients met with their care team bi-weekly during the first month of intervention and monthly thereafter; additional visits with different members of the team were scheduled as required (e.g., weekly to bi-weekly psychotherapy with psychologist). Notes from these visits were also uploaded to the hospital's electronic records for members of the patient's circle of care to access. Care plans were “living documents” that were individually tailored and evolved as the study progressed.

Patients enrolled in RIPAP were offered all services and specialized programs available at the Hospital Pain Centre (HPC) (e.g., medical management, including intervention if appropriate; CBT-based chronic pain management program; and regional opioid intervention services). Further, patients were provided with access to phone advice from an experienced nurse on the interdisciplinary team. Pain fellows, Acute Pain Services, and experienced HPC physicians worked together to ensure that patient care was optimized as rapidly as possible during admissions. Patients completed questionnaires at baseline and every 3 months during a 1-year follow-up.

### 2.5. Measures

#### 2.5.1. Primary Outcome

As recommended by IMMPACT [34], pain and functional impairment were measured using the Brief Pain Inventory–Short Form (BPI-SF [35]). The BPI-SF measures pain intensity, the impact of pain on seven daily activities (e.g., activity, work, and sleep), and analgesic use. The BPI was originally designed to measure cancer pain, but has been shown to be a reliable and valid instrument for measuring noncancer pain [36–39]. Test-retest values for pain and interference typically range between 0.72 and 0.98, and data from studies in many countries have supported a two-factor solution of pain severity and interference [40]. Studies quantifying the magnitude of improvement in pain and function that would constitute an important benefit to treatment of acute and chronic pain indicate that approximately 1-point reduction in pain or one-point improvement in interference represent minimally clinically significant change [41]. The primary outcomes in this study were average pain and functional impairment averaged across daily activities.

#### 2.5.2. Secondary Outcomes

Secondary outcomes included number of chronic pain-related visits to the ED and healthcare providers, emotional function, sleep disturbance, health-related quality of life, opioid risk, and global impression of change.

*(1) Chronic Pain-Related Visits to Healthcare Providers*. They were assessed using two methods. First, objectively measured visits to the ED in the previous 12 months was assessed at baseline and 12-month follow-up using the electronic medical records of the hospital. Second, during each visit, patients completed a self-report questionnaire reporting on the number of times they visited the ED, PCP, and specialists for chronic pain over the previous 3-month period.

*(2) Emotional Function*. Symptoms of depression were measured using the Patient Health Questionnaire-9 (PHQ-9 [42]), a self-report measure of the extent to which respondents have been bothered by 9 symptoms of depression, that correspond to DSM-IV criteria, over the past two weeks using a 4-point Likert scale from 0 “not at all” to 3 “nearly every day.” Scores range from 0 to 27 with scores of ≥5, ≥10, and ≥15, representing mild, moderate, and severe levels of depression severity [42], respectively. Psychometric properties of the PHQ-9 and sensitivity to change are well documented [43].

Symptoms of anxiety were measured using the Generalized Anxiety Disorder-7 (GAD-7 [44]), a self-report measure of the extent to which respondents have been bothered by 7 symptoms of generalized anxiety, that correspond to DSM-IV criteria, over the past two weeks using a 4-point Likert scale from 0 “not at all” to 3 “nearly every day.” Scores range from 0 to 21 with scores of ≥5, ≥10, and ≥15, representing mild, moderate, and severe levels of anxiety, respectively. Psychometric properties of the GAD-7 are well documented [43].

Anxiety related to pain was measured using the Pain Catastrophizing Scale (PCS [45]), a 13-item self-report questionnaire assessing how respondents think and feel when they experience pain using a 0 “not at all” to 4 “all the time” Likert scale. The PCS yields a total score and three subscale scores assessing rumination, magnification, and helplessness. Internal consistency of the PCS is excellent for the total score (Cronbach *α* = 0.93–0.95 in undergraduate, community, and outpatient pain samples) [45, 46] and good for subscale scores (Cronbach *α* = 0.75–0.95 in undergraduate and outpatient pain samples) [47].

*(3) Sleep Disturbance*. It was measured using the *Insomnia Severity Index* (ISI [48, 49]), a 7-item self-report questionnaire assessing the nature, severity, and impact of insomnia in the previous 2-weeks using a 5-point Likert scale from 0 “no problem” to 4 “very severe.” Scores range from 0 to 28 with scores of ≥8, ≥15, and ≥22, representing subthreshold, moderate, and severe levels of insomnia. Adequate psychometric properties have been reported in community samples, primary care patients, cancer patients, and chronic pain patients [50–52].

*(4) Opioid Risk*. It was assessed using the Screener and Opioid Assessment for Patients with Pain–Revised (SOAPP-R [53]), a 24-item self-report instrument used to determine risk potential for future aberrant drug-related behavior. Items are rated from 0 “never” to 4 “very often” using a Likert scale and summed to generate a total score from 0 to 96. Higher scores correlate with greater potential for future aberrant drug-related behavior. A cutoff score of 18 has been used to distinguish patients at risk of opioid abuse [53]. The SOAPP-R has good predictive validity, with an area under the curve of 0.88 [53]. The SOAPP-R is empirically derived. Support has been reported for internal reliability and predictive validity [53].

*(5) Health-Related Quality of Life*. It was measured using the *EuroQol* (EQ-5D-5L [54, 55]), a two-part instrument. Part 1 records self-reported problems on five domains (i.e., mobility self-care, usual activities, pain/discomfort, and anxiety/depression) using three levels of response (i.e., no problem, some problems, and extreme/severe problems). Participant scores were used to calculate an index value based on normative data from the United Kingdom [56]. Index scores range from 0 to 1 with scores of 1 reflecting optimal health and scores of 0 reflecting mortality. Part 2 asks the respondent to record their overall health using a 20 cm visual analogue scale (VAS) and can be interpreted directly as a quantitative measure of overall health.

*(6) Global Change*. Across the course of the study, global change was assessed using the *Patients' Global Impression of Change* scale (PGIC [57]). This measure is a single-item rating by participants of their improvement with treatment during a clinical trial on a 7-point scale ranging from “very much improved” to “very much worse” with “no change” as the midpoint. There has been wide use of the PGIC in chronic pain trials [58, 59], and data provide a responsive and readily interpretable measure of participants' assessment of clinical importance of treatment.

### 2.6. Statistical Procedures

Data were screened for potential outliers. No univariate outliers were identified using the recommended cutoff *z*-score of 3.29 [60]. Little's test for missing completely at random MCAR indicated that data were missing completely at random, *χ* ^2^ = 12.13, *p*=0.74. Missing data was handled using multiple imputation [61], with 10 imputations using all variables and all available data across time points. No variable met criteria for skewness or kurtosis as defined by a value in excess of 3.29 when values for skewness or kurtosis were divided by their respective standard errors [60].

Mean change from baseline to 12 months was calculated and tested for significance using paired-samples *t*-tests. Given the small sample size, no correction was performed to adjust for inflation of familywise error due to performing multiple *t*-tests. Standardized mean differences were calculated as a *Cohen's d* corrected for dependence among means using Morris and DeShon's equation [62]. A *d* of 0.41 was selected a priori to constitute a recommended minimum effect size representing a “practically” significant effect (refer to [63, 64]).

One purpose of this pilot study was to establish a proof of concept and obtain information about feasibility of recruitment and retention in preparation for a definitive study. We established our sample size based on resource availability and estimated a capacity to enroll 20 patients.

## 3. Results

**Figure 1:**
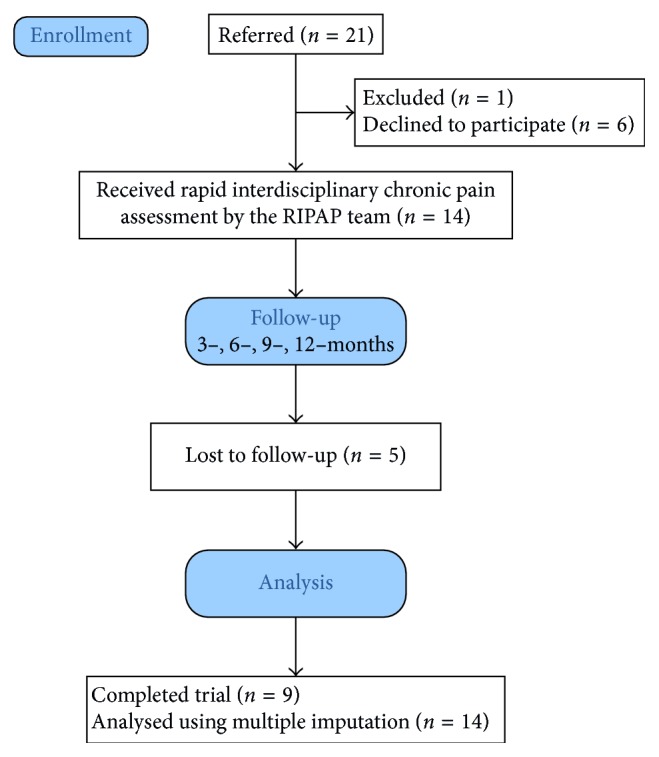
Study flow diagram.

Twenty-one patients with chronic pain were referred between January of 2014 and June of 2015; refer to Figure 1 for a study flow diagram. Six participants elected not to participate (i.e., scheduled but did not attend a baseline appointment or respond to three reminder telephone calls). One participant with active psychosis was excluded. The final sample consisted of 14 patients, 7 females, with a mean age of 36.2 years, SD = 15.7; refer to Table 1 for demographics.

Patients presented with a diverse range of chronic pain conditions, including neuropathic pain, temporomandibular joint pain, abdominal pain, complex regional pain syndrome, and pain related to inflammatory bowel disease. There was no particular pattern of comorbid medical conditions; we had patients with rare conditions (e.g., cystic fibrosis with double lung transplant; rare autoinflammatory conditions) as well as more common medical problems such as diabetes, hypertension, hyperlipidemia, and asthma. Patients had high healthcare utilization within the 12 months preceding baseline, with a mean 28.6, SD = 18.3, visits to the ED, and 1.2, SD = 1.5, inpatient hospital admissions lasting a mean 7.7, SD = 11.7, days. On average, patients reported moderately severe symptoms of depression, *M* = 12.43, SD = 7.25, mild symptoms of generalized anxiety, *M* = 9.28, SD = 6.41, and moderately severe symptoms of insomnia at baseline. Patients also scored at risk for future aberrant opioid use, *M* = 23.36, SD = 10.84. All patients had a primary care provider.

### 3.1. Attrition

Fourteen patients completed baseline assessment. Five of these patients did not complete the 12-month assessment: three (21%) patients discontinued participation following baseline assessment, one following 3-month assessment, and one following 6-month assessment. Nine patients completed baseline and 12-month assessments. Reasons for attrition included pregnancy (*n*=1), an unstable relationship with the team (*n*=1), and rupture in the relationship with the team (or a team member) relative to prescription opioids (*n*=3).

In addition, not all patients engaged equally with different services offered, even when those where recommended. However, our sample size was too small to explore if this had an impact on outcome. We have provided the number of patients who visited various providers of the program and hospital based services in Supplemental Table 1.

### 3.2. Change from Baseline to 12 Months

Table 2 presents change in outcome variables from baseline to 12 months (the primary endpoint of this study). Statistically and practically significant improvements from baseline to 12 months were observed for pain interference, average 24-hour pain, symptoms of depression, symptoms of insomnia, pain catastrophizing, risk of future aberrant opioid behavior, health-related quality of life, self-reported health, self-reported medical visits, and objectively measured ED visits.

On average, per annum ED visits were reduced from 28.64 at baseline to 5.14 at 12-month follow-up, *M*_Diff_ = −23.50 (95% CI: −12.77, −34.23), with all but one patient experiencing a reduction in ED visits of more than 50%. Similarly, mean reductions of more than 1-point were observed for pain interference, *M*_Diff_ = −1.41 (95% CI: −0.07, −2.74), and average 24-hour pain, *M*_Diff_ = −1.97 (95% CI: −0.76, −3.18), from baseline to 12 months. Five (56%) of the nine patients who completed the study reported a mean reduction in pain interference greater than 1 point, and six (67%) reported a mean reduction in average 24-hour pain that exceeded 1 point. The mean reduction in symptoms of depression was 5.12 (95% CI: −1.54, −8.70) points with all but one patient reporting fewer symptoms of depression at 12-month follow-up relative to baseline. Eight of the nine patients who completed the study reported a reduction in risk of future aberrant opioid use with a mean reduction of 7.53 (95% CI: −3.24, −11.80) points at 12 months (Table 2).

Of the 9 patients who completed the 12-month assessment, 1 patient rated their change as “somewhat better, but the change has not made any real difference,” 3 as “moderately better, and a slight but noticeable change,” 2 as “better, and a definite improvement that has made a real and worthwhile difference,” and 3 as “a great deal better, and a considerable improvement that has made all the difference,” on the PGIC scale.

Change in outcome variables across baseline, 3-, 6-, 9-, and 12-month measurements have been depicted in Supplemental Table 2 to serve as a heuristic for the development and design of future studies.

## 4. Discussion

This prospective cohort study evaluated the effect of a care-plan intervention on healthcare visits and clinical outcomes among patients with chronic pain. High frequency visitors of the ED (i.e., patients with >12 ED visits in the previous year, of which ≥50% were related to chronic pain) were identified and provided with rapid access to a comprehensive interdisciplinary assessment and the development and implementation of an individualized chronic pain care plan. Patients were assessed at 3-month intervals over a 12-month follow-up period. The intervention resulted in clinically significant improvements in ED visits, medical visits, pain, physical and emotional function, sleep, and risk for future aberrant opioid use.

Rapid interdisciplinary chronic pain assessment and development of a patient-specific care plan reduced self-reported and objective indices of chronic pain-related healthcare utilization by more than 20 visits (>80%) per year from pre- to postintervention. A recent review of prospective cohort trials evaluating the effect of interventions on ED visits among high frequency visitors with varied health conditions reported a mean reduction of 1.91 visits per year across 10 studies, though significant variability was noted between studies (range from +2.79 to −37) [27]. Previous reviews have reported a positive association between intervention intensity (e.g., frequency of follow-up and availability of psychosocial resources) and strength of outcomes [25]. Further, multimodal case management and care-plan interventions that have included uploading information to an electronic information sharing system that is accessible to other healthcare providers [65, 66] have shown the strongest intervention effects [27]. Consistent with these observations, we employed an intensive multimodal intervention and observed large effects on chronic pain-related ED visits as well as self-reported medical visits. Importantly, this represents the first prospective cohort study to demonstrate that a care-plan intervention can be effectively used to reduce chronic pain-related ED visits and improve clinical outcomes.

Intervention effects were evaluated across the core clinical outcomes important for chronic pain trials as recommended by the IMMPACT task force [34]. Patients reported practically significant improvements on their average, worst, and present pain, as well as function from pre- to postintervention. Reductions in worst, average, and present pain, as well as function, exceeded the recommended criteria for minimally clinically significant improvement of 1-point on an 11-point numeric rating scale [41]. Moreover, the intervention resulted in concomitant improvements in depressed mood, insomnia severity, pain catastrophizing, health-related quality of life, and self-reported health. Reductions in symptoms of depression were observed in all but one patient who completed the study, with 5/9 patients reporting clinically significant improvements, defined as a 50% reduction and a posttreatment score < 9 [42, 67]. The mean reduction in symptoms of insomnia fell between slight and moderate clinically significant improvement [51]. The overall pattern of results suggests that the intervention improved patient function and psychological pain tolerance [68, 69].

Results from the present study suggest that rapid interdisciplinary chronic pain assessment and development of an individualized care plan can reduce the risk of opioid misuse. Analysis of patient reported SOAPP-R data indicated clinically relevant improvements in risk of future aberrant opioid use. On average, patients were at high risk (scores ≥ 22) of opioid misuse at baseline and moderate risk (scores 10–21) at 12-month follow-up [53]. Importantly, 7/9 patients reported improvement in risk of future aberrant opioid use at 12-month follow-up and only 2/9 reported no change in risk of future aberrant opioid use. This result has important implications for addressing the burden associated with opioid misuse. Overdose deaths involving opioid analgesics have risen sharply over the past two decades. The rate of opioid deaths in the United States increased from 4,030 in 1999 to 14,800 in 2008 [70], and the rate in Ontario increased by 242% between 1991 (12.2 per 1,000,000) and 2010 (41.6 per 1,000,000) [71]. Data from the United States indicated that a significant portion (between 15% and 30%) of the 201.9 million opioid prescriptions dispensed in 2009 were prescribed in the ED [72, 73], a location often targeted by patients seeking opioid prescriptions for nonmedical use [74]. We expect that by implementing individualized care plans and ensuring supportive follow-up, healthcare providers can aid in abating the risk of opioid misuse, abuse, and morbidity among patients with chronic pain.

Several patients experienced a ruptured relationship with the team regarding opioid prescribing. Three patients were not ready to reduce their opioid use despite efforts made by the team to engage patients in open and nonjudgmental conversations about the risk and benefits of opioid use for chronic noncancer pain and offer opioid substitution as well as agents to mitigate symptoms of withdrawal in case an opioid wean was indicated. This was highlighted by two patients who described feeling as though they were treated as “drug seekers” in the independent program evaluation. Better attention to the team's approach may be needed to effectively work with patients on opioids for whom a different pain management strategy is required due to the high risk of harm, limited benefits observed, or signs of opioid aberrancies.

This study has important implications for public health. Chronic pain is rated by family physicians as the second most difficult condition to treat after mental health [75, 76], and the two often cooccur [77]. Patients with chronic pain who have unmet needs will turn to different sources to find relief, including the ED. The ED is not the appropriate setting to treat chronic pain as it is associated with an increase in risk of adverse events or mortality [5, 20–22]. For example, Dhalla et al. [22] reported that 66.4% of patients who died of an opioid overdose saw a healthcare provider (family physician or ED attendant) within the month that preceded their death, of whom 56.1% filled a prescription for an opioid, often prescribed for pain-related complaints. In the context of the unprecedented opioid crisis affecting Canada, United States, and countries around the world, there is a clear need for evidence-based approaches to manage chronic pain that do not involve the use of opioids. We demonstrated that an interdisciplinary program, that included care plans in the case of ED visits along with detailed recommendations for pharmacological, interventional (if appropriate), and nonmedicinal approaches, improved clinical outcomes and reduced visits to the ED among patients presenting primarily with chronic pain. This approach can be adapted to any hospital with dedicated pain services or with linkages to community-based interdisciplinary pain programs and might help mitigate the adverse effects associated with the opioid crisis.

### 4.1. Limitations

The results of this prospective cohort study must be interpreted with caution due to several limitations. First, a single-sample prospective cohort design was employed. Without an adequate control group, it was not possible to control for confounds, such as selection bias, expectation, increased attention, or maturation. Further, attrition appears to be a hallmark of frequent ED use [2], with frequency of ED visits among HFVs often declining from one year to the next [78]. It is impossible to account for such natural decline in frequent ED use from year to year without the use of a control sample. Second, the sample size was small with a modest level of attrition (36%). While concerns over attrition were partially remedied using multiple imputation, the small sample size raises uncertainty about the reliability of the observed effects. Small samples can produce false-positive results or overestimate the magnitude of effect [79]. Further, studies with small sample sizes make generalizability of results particularly challenging given that there is a lower likelihood of capturing a sample that generalizes to the major variants typically observed in the population sample (in this case HFVs of the ED who present with chronic pain). Similarly, three patients withdrew from the study due to ruptured relationships with the team over opioid prescribing, suggesting that data might not have been missing completely at random. It should be noted that risk of future aberrant opioid use was included as a modeled variable in multiple imputation. Owing to these considerations, care should be taken and thoughtful consideration should be made when extrapolating the results of this study to other populations and settings. Third, while global improvements were observed, this was an intensive interdisciplinary intervention that resulted in an increase in cost of care. This increase in direct costs of care may, however, be offset by improvements in ED utilization and overcrowding and in staff and patient satisfaction. Fourth, ED visits were measured using the hospital's electronic medical record system which does not capture ED visits at hospitals outside of hospital system. It should be noted that patients self-reported similar reductions in healthcare visits and that these self-reports should capture all sources of healthcare utilized. Finally, as with any multimodal treatment, it is unclear which aspects of the intervention were most valuable or effective. Future research is required to determine which elements of treatment are driving the observed effects, and should include investigation into specific characteristics of patients who engage with, and benefit from rapid interdisciplinary intervention.

## 5. Conclusion

An intervention consisting of rapid interdisciplinary assessment and development of an individualized care plan targeting high frequency users of the ED with chronic pain may be effective at reducing ED use and improving clinical outcomes. Rapid assessments and individualized care plans uploaded to an electronic medical record system may be worth implementing in hospital EDs for high frequency visitors with chronic pain. Additional methodologically rigorous randomized controlled trials are needed to confirm the beneficial effects observed in this study.

## Figures and Tables

**Table 1:** Demographic characteristics.

Characteristic	Total	
	No.	%
Age (years)		
19–29	7	50.0
30–39	4	28.6
40–49	2	14.3
50+	1	7.1
Ethnicity		
White	10	71.4
East Indian	1	7.1
Mixed	3	21.4
Duration of chronic pain (years)		
1–5	6	42.8
6–10	4	28.6
11–15	1	7.1
15+	3	21.4
Marital status		
Single	7	50.0
Married	5	35.7
Common law	1	7.1
Widowed/widower	1	7.1
Employment status		
Unemployed	2	14.3
Social assistance	5	35.7
Full time	2	14.3
Student	3	21.4
Retired	2	14.3

*Note. N* = 14; 7 females.

**Table 2:** Change in pain and psychosocial function from baseline to 12 months.

Variable	Baseline *M* (SD)	12-months *M* (SD)	*M*_Diff_ 95% (LCI: UCI)	*t*-value	Cohen *d*	Effect size convention^1^
Pain interference	6.09 (2.51)	4.68 (2.41)	−1.41 (−0.07: −2.74)	2.06^∗^	0.71	Minimum PSE
Worst pain last 24-hours	7.71 (2.09)	6.02 (2.14)	−1.69 (−0.12: −3.28)	2.18^∗^	0.74	Minimum PSE
Least pain last 24 hours	3.64 (2.68)	3.12 (2.04)	−0.52 (0.92: −1.96)	0.71	0.31	—
Average pain last 24 hours	5.64 (2.09)	3.67 (2.32)	−1.97 (−0.76: −3.18)	3.28^∗∗^	1.31	Medium PSE
Pain right now	5.71 (2.13)	4.53 (2.24)	−1.18 (0.14: −2.50)	1.76^†^	0.69	Minimum PSE
Anxiety	9.28 (6.41)	5.46 (3.42)	−3.82 (0.70: −8.34)	1.66^†^	0.45	Minimum PSE
Depressed mood	12.43 (7.25)	7.31 (3.01)	−5.12 (−1.54: −8.70)	2.80^∗∗^	0.98	Minimum PSE
Insomnia severity	16.86 (5.11)	11.40 (5.38)	−5.46 (−2.28: −8.64)	3.37^∗∗^	1.00	Minimum PSE
Health-related quality of life	0.65 (0.07)	0.74 (0.08)	0.14 (0.09: 0.18)	3.15^∗∗^	1.74	Medium PSE
Self-report health	4.17 (1.91)	6.02 (1.18)	−1.85 (−2.98: −0.72)	3.21^∗∗^	0.98	Minimum PSE
Pain catastrophizing	29.86 (11.73)	14.18 (7.41)	−15.68 (−6.89: −24.46)	3.50^∗∗^	0.91	Minimum PSE
SOAPP-R	23.36 (10.84)	15.83 (5.97)	−7.53 (−3.24: −11.80)	3.45^∗∗^	1.49	Medium PSE
Self-report medical visits in past 3 months for chronic pain	8.43 (5.37)	3.40 (2.09)	−5.03 (−2.10: −7.95)	3.37^∗∗^	1.09	Minimum PSE
ED visits in past 12 months	28.64 (18.32)	5.14 (7.10)	−23.50 (−12.77: −34.23)	4.29^∗∗^	1.23	Medium PSE

*Note*. *N* = 14; df = 13; 7 females; ^†^ *p* < 0.10; ^∗^ *p* < 0.05; ^∗∗^ *p* < 0.01; ED = emergency department; *M*_Diff_ = mean difference; PE = practically significant effect; PGIC = Patients' Global Impression of Change; SOAPP-R = Screener and Opioid Assessment for Patients with Pain–Revised; ^1^effect size conventions for clinicians and researchers.

## Supplementary Materials

Supplementary Materials Supplemental Table 1: Number of health care provider visits associated with CIPAP program. Supplemental Table 2: Change in pain and psychosocial function from baseline to 3-, 6-, 9-, and 12-months.
