# Supplementary material for: Chronic Pain in the Emergency Department: A Pilot Interdisciplinary Program Demonstrates Improvements in Disability, Psychosocial Function, and Healthcare Utilization
Source: Pain Res Manag. 2018 Jan 17;2018:1875967. doi: 10.1155/2018/1875967 (PMC5829435; doi:10.1155/2018/1875967)
Supplement: Supplementary Materials — Supplemental Table 1: Number of health care provider visits associated with CIPAP program. Supplemental Table 2: Change in pain and psychosocial function from baseline to 3-, 6-, 9-, and 12-months. [file 1875967.f1.docx]

Supplemental Material

Sample Care Plan

**Familiar Faces in ED Chronic Pain Program Treatment Plan**

Institution Logo

**Service Date: Name/Nom:**

**Unit/Clinic: Medical Record #**

**Attending: DOB/DDN Gender:**

**Visit Type: Admission/ Visit Date:**

**Campus: Discharge Date:**

**Please Respect Patient Confidentiality**

***This patient is registered in a program for patients suffering with chronic pain and visiting the emergency department frequently (FFCP). Please see our recommendations below for ED and outpatient care.***

Ms. X is a XYZ woman presenting with chronic lower abdominal/suprapubic/lower back pain; pain is associated with nausea. She reported pain since XXXX date when ureteropelvic junction obstruction was diagnosed and right kidney removed. Past medical history also includes: anxiety, depression, dysuria, suspected irritable bowel syndrome.

Ms. X has visited the ED 20 times from September to November 2016; 5 visits in October were noted for dental pain following abscess/tooth extraction and others were for chronic abdominal pain.

The treatment plan has been discussed by an interdisciplinary team: (Team members and discipline listed). Case manager is XXX.

The following is a summary of the treatment plan devised following the outpatient pain case conference and agreed upon by the patient.

**Emergency Department:**

1. Acute healthcare problems should be investigated and managed as per usual standard in the ED. If appropriate, education regarding chronic pain and reassurance that medical problems have been ruled out.
2. **Opioid Risk Score is High**, therefore we advise against the use of opioids in the ED.
3. Outpatient management of chronic unexplained pain is to be managed through the Pain Clinic, therefore please re-direct her to our outpatient program. Should she present during regular business hours, please do not hesitate to contact the pain clinic and we will see her on an urgent basis.

**Outpatient Plan:**

In the short term, the FFCP team will:

1. Liaise with family physician (to discuss care and any aberrant drug-related behaviours).
2. Consent to release and obtain information (Dr. XX, family doctor, community physio, mobile crisis team, hospital units, pharmacy).
3. Social Work (home visit, will discuss social assistance application process).
4. Physiotherapy (home exercise, walking program).
5. Occupational therapy (sense of purpose, pacing, work into usual activities slowly such as grocery shopping)
6. Further assessment with Psychology (given presentation, r/o personality/dissociative identity disorder; introduce coping strategies for pain, anxiety, and depression).
7. Support from our addiction specialist is available but patient is reluctant to engage.
8. Education about what to look for indicating that solitary kidney is being harmed and reassure that pain, especially when chronic, does not indicate a serious medical problem.
9. Pain Clinic Education Sessions (pain psychoeducation).
10. Avoid NSAIDs completely.
11. Avoid TCAs because of suicidality and history of urinary retention.
12. Consider reducing duloxetine to 90 mg and then to 60 mg.
13. Increase pregabalin to 25 mg BID and 100 mg qhs.
14. Consider nabilone qhs for sleep/pain/anxiety.

Revisions to this plan (as appropriate) and update on progress will be provided following each visit.

Sincerely,

Supplemental Table 1

*Number of Health Care Provider Visits Associated with CIPAP Program*

| Health Care Provider | # of Patients who Accessed Service | # of Visits |
| --- | --- | --- |
| Pain Medicine (Anesthesiology) | 14 | 165 |
| Psychology | 13 | 64 |
| Addiction Specialist | 4 | 4 |
| Social Work | 5 | 10 |
| Other Services (e.g., surgery, orthopedic, gastroenterology, neurology) | 12 | 61 |

Supplemental Table 2

*Change in Pain and Psychosocial Function from Baseline to 3-, 6-, 9-, and 12-months*

| Variable | Baseline  M (SD) | 3-months  M (SD) | 6-months  M (SD) | 9-months  M (SD) | 12-months  M (SD) |
| --- | --- | --- | --- | --- | --- |
| Pain interference | 6.09 (2.51) | 5.68 (2.57) | 5.18 (2.56) | 4.43 (2.45)* | 4.68 (2.41)* |
| Worst pain last 24-hours | 7.71 (2.09) | 7.72 (2.44) | 6.79 (2.98) | 5.10 (2.69)** | 6.02 (2.14)* |
| Least pain last 24-hours | 3.64 (2.68) | 4.10 (1.93) | 3.61 (2.25) | 2.58 (2.24) | 3.12 (2.04) |
| Average pain last 24-hours | 5.64 (2.09) | 5.33 (2.12) | 4.64 (2.13) | 3.40 (2.33)** | 3.67 (2.32)** |
| Pain right now | 5.71 (2.13) | 6.46 (2.13) | 4.79 (2.61) | 4.21 (2.88) | 4.53 (2.24)† |
| Anxiety | 9.28 (6.41) | 6.50 (5.09) | 7.39 (4.47) | 5.70 (3.53) | 5.46 (3.42)† |
| Depressed mood | 12.43 (7.25) | 10.19 (7.03) | 9.17 (5.27)† | 5.99 (4.15)** | 7.31 (3.01)** |
| Insomnia severity | 16.86 (5.11) | 14.86 (3.89) | 12.48 (4.30)† | 9.99 (5.05)** | 11.40 (5.38)** |
| Health-related quality of life | 0.65 (.07) | 0.66 (.07) | 0.68 (.09) | 0.70 (.08)† | 0.74 (.08)** |
| Self-report health | 4.17 (1.91) | 4.79 (1.63) | 4.58 (2.36) | 5.54 (1.70)* | 6.02 (1.18)** |
| Pain catastrophizing | 29.86 (11.73) | 22.89 (9.86)† | 21.49 (11.25)* | 17.42 (9.51)** | 14.18 (7.41)** |
| SOAPP-R | 23.36 (10.84) | 19.05 (8.71) | 19.36 (9.19) | 16.44 (7.67)** | 15.83 (5.97)** |
| Self-Report Medical visits in past 3-months for chronic pain | 8.43 (5.37) | 5.38 (2.80)** | 3.62 (2.11)** | 1.90 (0.72)** | 3.40 (2.09)** |
| ED Visits in Past 12-Months | 28.64 (18.32) |  |  |  | 5.14 (7.10)** |

Note. N = 14; *df* = 13; 7 female; † = *p*< .10; * = *p*< .05; ** = *p*< .01. All statistical comparisons were made relative to baseline. ED = Emergency Department; M_Diff_ = Mean Difference; PE = Practically Significant Effect; PGIC = Patient Global Impression of Change; SOAPP-R = Screener and Opioid Assessment for Patients with Pain - Revised. ^1^ Effect size conventions for clinicians and researchers.
